# Thermostability improvement of a *Talaromyces leycettanus* xylanase by rational protein engineering

**DOI:** 10.1038/s41598-017-12659-y

**Published:** 2017-11-10

**Authors:** Xiaoyu Wang, Rui Ma, Xiangming Xie, Weina Liu, Tao Tu, Fei Zheng, Shuai You, Jianzhong Ge, Huifang Xie, Bin Yao, Huiying Luo

**Affiliations:** 1 Key Laboratory for Feed Biotechnology of the Ministry of Agriculture, Feed Research Institute, Chinese Academy of Agricultural Sciences, Beijing, 100081 People’s Republic of China; 2 College of Biological Sciences and Biotechnology, Beijing Forestry University, Beijing, 100083 People’s Republic of China

## Abstract

Thermophilic xylanases with high catalytic efficiency are of great interest in the biofuel, food and feed industries. This study identified a GH11 xylanase gene, *Tlxyn11B*, in *Talaromyces leycettanus* JCM12802. Recombinant *Tl*Xyn11B produced in *Pichia pastoris* is distinguished by high specific activity (8259 ± 32 U/mg with beechwood xylan as substrate) and excellent pH stability (from 1.0 to 10.5). The beechwood xylan hydrolysates consisted mainly of xylobiose, xylotriose and xylotetraose, thus *Tl*Xyn11B could be used for the production of prebiotic xylooligosaccharide. By using the structure-based rational approach, the N-terminal sequence of *Tl*Xyn11B was modified for thermostability improvement. Mutants S3F and S3F/D35V/I/Q/M had elevated *T*_m_ values of 60.01 to 67.84 °C, with S3F/D35I the greatest. Homology modeling and molecular dynamics (MD) simulation analysis revealed that the substituted F3 and I35 formed a sandwich structure with S45 and T47, which may enhance the overall structure rigidity with lowered RMSD values. This study verifies the efficiency of rational approach in thermostability improvement and provides a xylanase candidate of GH11 with great commercialization potential.

## Introduction

Xylan accounts for approximately 35% of the dry weight of plant cell wall and represents one of the most important, abundant, and renewable bioresources. It is cross-linked with lignin and cellulose, which hinders it from efficient utilization. Due to the complex structure, its complete degradation requires the synergistic action of xylanolytic enzymes including endo-1,4-β-xylanase (EC 3.2.1.8), 1,4-β-xylosidase (EC 3.2.1.37), α-glucosiduronase (EC 3.2.1.139), α-L-arabinofuranosidase (EC 3.2.1.55), acetyl esterase (EC 3.1.1.72), ferulic acid esterase (EC 3.1.1.73), and p-coumaric acid esterase (EC 3.1.1.x)^1^. Endo-1,4-β-xylanase as the key enzyme decomposes inherent heteroxylans into short oligosaccharides or (and) xylose by randomly attacking the β-1,4-linkage in the xylan backbone^2^, and assists cellulase to access cellulose.

Xylanases have been widely applied in many fields, such as pharmacy, food and feed industry, biofule, textile, etc^3^. According to the sequence identity and three-dimensional structure homology, xylanases are classified into glycoside hydrolase (GH) families 5, 8, 10, 11 and 30 (http://www.cazy.org/), with the majority in GH10 and 11^3,4^. GH10 xylanases have typical triosephosphate isomerase (TIM) structures with greater molecular mass, while those from GH11 share a right hand β-jelly roll structure and are industrially attractive due to the small size, high substrate selectivity, and favorable pH and temperature optima^3^.

To meet the industrial demands, two protein engineering approaches, i.e. directed evolution and rational design, are commonly used to improve enzyme properties^5,6^. The directed evolution is a robust strategy to engineer enzymes by accelerating protein evolution^7^. However, this method involves in construction of large libraries and laborious screening. In contrast, the rational design derived from previous studies and sequence analysis is less laborious and time consuming^8^. Recently, computer-aided approaches have been used to analyze the primary sequence, simulate the tertiary structure and *de novo* predict the protein interaction^9^. Based on these bioinformatic analyses, key amino acids are identified and site-directed mutagenesis is conducted. This *in silico* rational method thus dramatically reduces the library size and workload and increases the engineering efficiency as well, and is widely used for protein improvement.

A large number of GH11 xylanases have been cloned from fungi and bacteria and biochemically characterized^10–12^. However, their wide applications have been limited by the weak thermostability. It is found that the N-terminal sequence plays a key role in the stability of GH11 xylanases. By replacing the N-terminus with thermostable ones^13,14^, introducing a disulfide bond to the N-terminus^15^, substituting the N-terminal residues^16^, and optimizing the surface charge interactions^17^, the thermostability of GH11 xylanases have been successfully improved. The underlying mechanism has been ascribed to the more amino acid interactions and decreased unfolding entropy^13^. In this study, a highly active GH11 xylanase with a broad pH adaptability range but weak thermostability was identified in *Talaromyces leycettanus* JCM12802. To improve its thermostability, a rational design focused on the N-terminal region was conducted. As results, a mutant with greater thermostability was obtained, and the underlying mechanism was then revealed by the analysis of molecular dynamics simulation.

## Results

### Gene cloning and sequence analysis

The full-length cDNA of *Tlxyn11B* (GenBank accession No. KY594261) contains 654 bp that encodes a 217 amino acid-residue polypeptide. The N-terminal 19 amino acid residues were predicted to be a putative signal peptide, and the mature protein was predicted to have a calculated molecular mass of 21.9 kDa and a *p*I of 4.63. The deduced amino acid sequence of *Tl*Xyn11B shares the highest identity of 84% with the xylanase A of *Penicillium* sp. 40, and of 73% with the structure-resolved GH11 endo-1,4-β-xylanase from *Talaromyces cellulolyticus* (PDB: 3WP3). By using the crystal structure 3WP3 as template, modeled *Tl*Xyn11B showed a β-jelly roll structure of typical GH11 xylanases and an extended N-terminus at the bottom of the palm region (Supplementary Fig. 1). Based on the Hotspot Wizard analysis, two conserved glutamates (94 and 185) were suggested to be catalytic residues. Energy minimization analysis of modeled *Tl*Xyn11B by Chimera 1.10 (http://www.cgl.ucsf.edu/chimera/) with steepest decent methods and conjugate gradient methods indicated that the conformation of this N-terminus has no tremendous changes before and after energy minimization. Thus we conjectured that certain interactions occurred in the N-terminus of *Tl*Xyn11B, which probably contribute to the fixation of this local structure at the bottom of the palm region.

### Production and characterization of the recombinant *Tl*Xyn11B

The DNA fragment coding for the mature *Tl*Xyn11B without the signal peptide sequence was successfully expressed in *P. pastoris* GS115. The culture supernatants of the positive transformant with highest xylanase activity were then purified to electrophoretic homogeneity as shown in Supplementary Fig. 2. The protein concentrate was determined to be 384 μg/mL, and the recovery rate was 48% (Supplementary Table 1). Asn75 as the only potential *N*-glycosylation site of *Tl*Xyn11B might link glycans during heterologous expression in *P. pastoris*. With and without the Endo H treatment to remove *N*-linked glycans, the purified enzymes migrated two single bands of almost similar apparent molecular weights (approximately 22 kDa), which are in agreement with the calculated value of *Tl*Xyn11B (21.9 kDa). This result indicated that no or little N-glycosylation occurred in *Tl*Xyn11B. Further mass (MS) analysis verified the identity of the recombinant proteins as *Tl*Xyn11B, since the polypeptide sequences of the target band were consistent with the sequence of deduced *Tl*Xyn11B (Supplementary Fig. 3).

**Figure 1:**
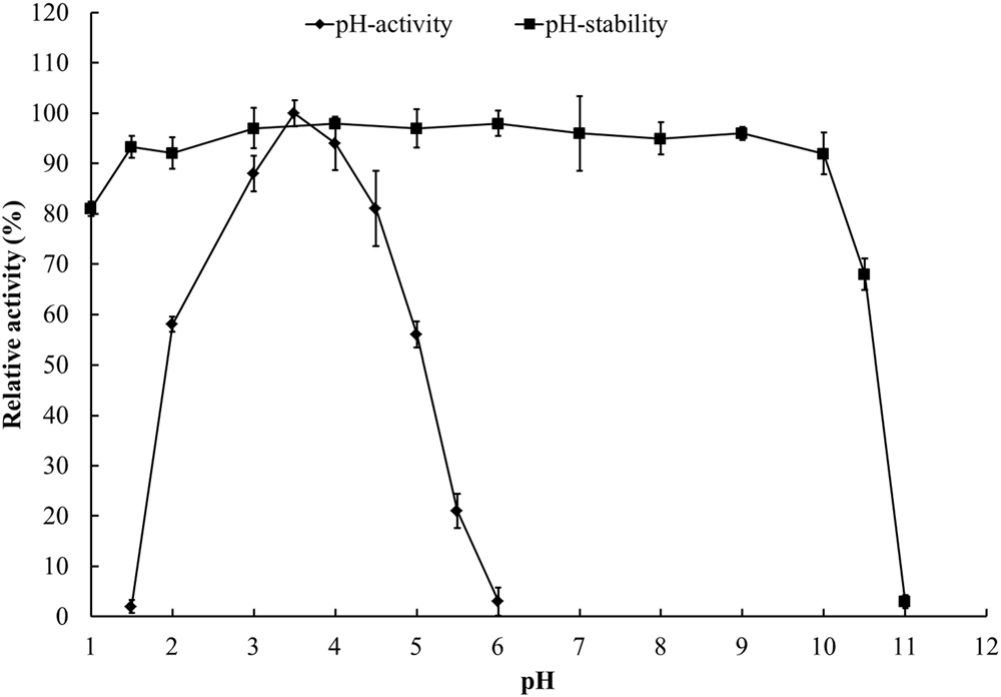
pH properties of the purified recombinant *Tl*Xyn11B.

**Figure 2:**
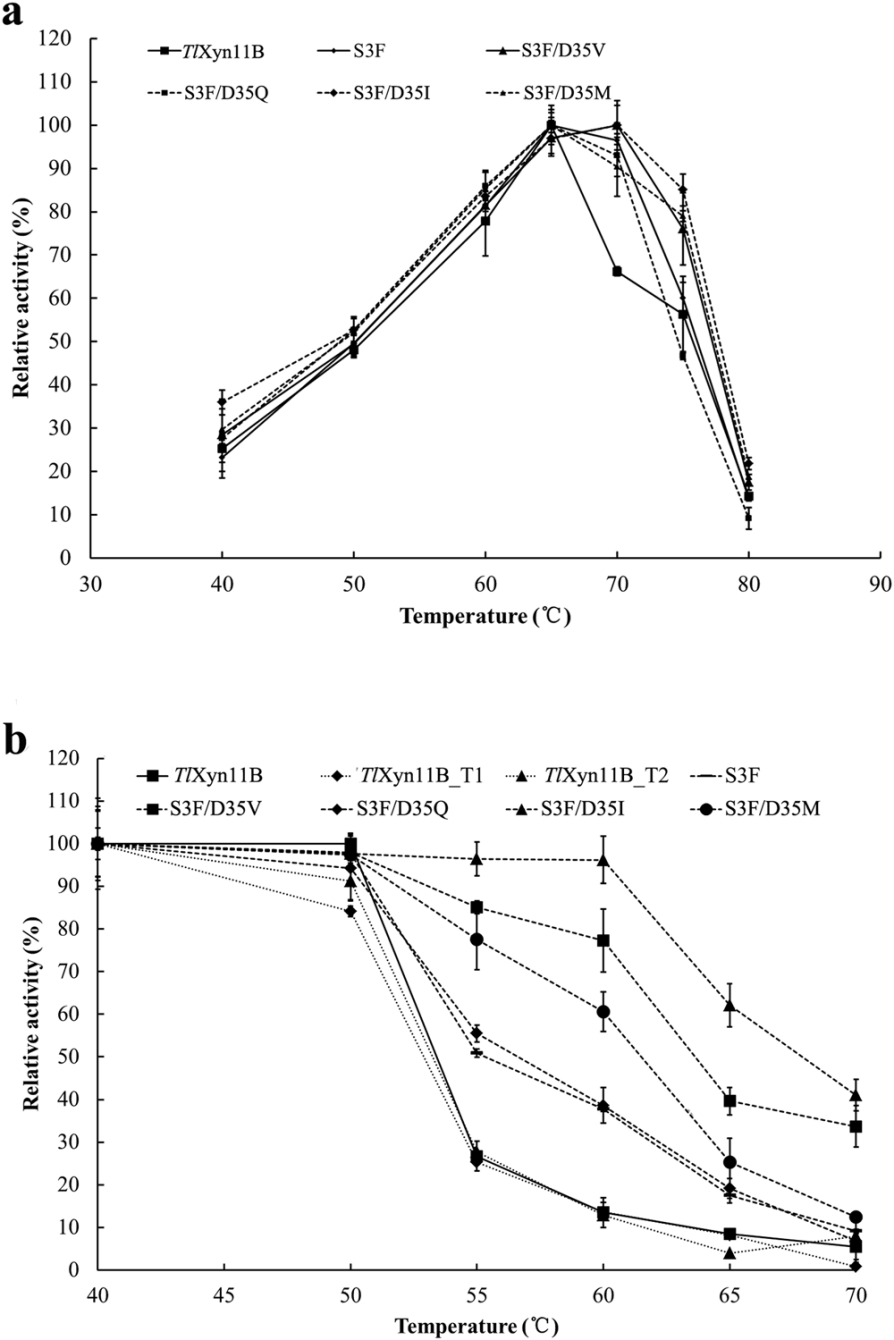
Thermal properties of the purified recombinant *Tl*Xyn11B and its mutants. (**a**) Effect of temperature on the xylanase activities. (**b**) Thermostability after 30 min-incubation.

*Tl*Xyn11B was acidophilic, exhibiting maximum activity at pH 3.5 and remaining more than 50% activity over the acidic pH range (pH 2.0–5.0) (Fig. 1). After incubation at 37 °C for 1 h, the enzyme showed great stability over a broad pH range of 1.0–10.5, retaining more than 80% activity at pH 1.0–10.0 and 68% at pH 10.5 of the initial activity (Fig. 1). It had a temperature optimum of 65 °C (Fig. 2a), but retained stability at 50 °C and below (Fig. 2b). Different concentrations (1–10 mM) of metal ions and chemical reagents demonstrated distinct effects on *Tl*Xyn11B activity (Table 1). The enzyme displayed strong resistance to all tested chemicals at 1 mM, but was sensitive to Pb^2+^, Mn^2+^, Cu^2+^, Fe^3+^ and SDS at the concentration of 5 mM or (and) 10 mM. Using beechwood xylan as the substrate, the specific activity, *V*_*max*_, *K*_*m*_, and *k*_*cat*_/*K*_*m*_ of *Tl*Xyn11B were determined to be 8,300 ± 32 U/mg, 12,800 ± 42 U/mg, 3.3 ± 0.6 mg/mL, and 1,420 ± 26 mL/s/mg, respectively.

**Table 1:** Effects of metal ions or chemical reagents on *Tl*Xyn11B activity.

Chemicals	Relative activity (%)^a^		
	1 mM	5 mM	10 mM
Control	100.0 ± 2.2	100.0 ± 1.7	100.0 ± 3.1
K^+^	103.2 ± 1.1	112.8 ± 1.7	116.3 ± 4.4
Mg^2+^	99.4 ± 2.4	106.9 ± 4.0	101.7 ± 2.7
Na^+^	101.4 ± 1.5	107.5 ± 12.3	105.5 ± 7.5
Ca^2+^	100.8 ± 3.3	109.3 ± 0.9	104.6 ± 4.2
Pb^2+^	92.1 ± 4.6	86.5 ± 2.3	58.2 ± 5.6
Mn^2+^	96.3 ± 3.2	82.9 ± 0.9	64.2 ± 10.7
Ni^2+^	102.6 ± 1.3	107.8 ± 0.4	98.2 ± 2.4
Cr^3+^	105.4 ± 3.8	101.8 ± 5.9	96.4 ± 4.5
Cu^2+^	90.9 ± 5.2	85.6 ± 8.6	68.1 ± 3.7
Zn^2+^	101.3 ± 7.1	99.9 ± 1.2	96.4 ± 0.3
Fe^3+^	88.4 ± 3.6	54.7 ± 1.3	22.3 ± 5.2
EDTA	104.8 ± 6.3	113.3 ± 8.9	106.6 ± 5.8
SDS	73.6 ± 2.5	33.1 ± 5.6	11.5 ± 3.7

^a^Data are shown as mean** ± **SD (n = 3).

**Figure 3:**
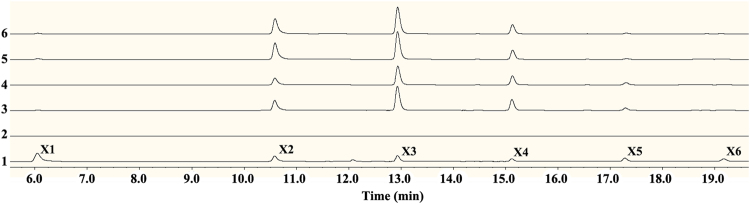
HPAEC-PAD analysis of the end products. 1 Standards: X1, xylose; X2, xylobiose; X3, xylotriose; X4, xylotetraose; X5, xylopentaose; and X6, xylohexaose. 2 Background. 3 Products of *Tl*Xyn11B. 4 Products of S3F. 5 Products of S3F/D35V. 6 Products of S3F/D35I.

The hydrolysis products of beechwood xylan by *Tl*Xyn11B consisted mainly of xylobiose (25%), xylotriose (42%) and xylotetrose (29%) (Fig. 3). This result was in agreement with previous publications^18^ that xylotriose is predominant in the hydrolysates by GH11 xylanases.

### Characterization of mutant enzymes

S3 and D35 were selected for mutation due to their high B-factor values and special positions. The former is located at the N-terminus, which has been verified to affect thermostability^19^, while the latter located on the bottom of a palm region tends to expose in the solution and interact with the N-terminus. Saturated mutation was conducted at residues S3 and D35 sequentially. The first round screening indicated that mutant S3F had enhanced thermostability. Based on this, saturated mutation at D35 in combination with S3F was performed. And four double mutants, i.e. S3F/D35V, S3F/D35Q, S3F/D35I and S3F/D35M, showed improved thermostability.

The catalytic and thermal properties of the wild type and mutant enzymes S3F and S3F/D35V/Q/I/M were then compared. Interestingly, all mutants showed improved thermostability without activity reduction. With beechwood xylan as the substrate, all mutants had specific activities comparable to or greater than the wild type (Table 2). In terms of temperature optima, those of S3F/D35I and S3F/D35V shifted to 70 °C while the others remained 65 °C as the wild type (Fig. 2a). After incubation at 40 to 70 °C for 30 min, all mutants showed improved thermostability (Fig. 2b). Of them, S3F/D35I was the most thermostable, followed by S3F/D35V. DSC results confirmed the above results (Table 2). The *T*_m_ of wild type was 60.0 °C, which was 2.3, 4.7, 5.8, 6.6 and 7.8 °C lower than those of mutant S3F, S3F/D35Q, S3F/D35M, S3F/D35V and S3F/D35I, respectively.

**Table 2:** The specific activities and *T*_m_ values of *Tl*Xyn11B and its mutants.

Enzyme	Specific activity (U/mg)	*T*_m_ (°C)
*Tl*Xyn11B	8,400 ± 43	60.0
S3F	9,300 ± 62	62.3
S3F/D35Q	9,000 ± 24	64.7
S3F/D35M	9,100 ± 73	65.8
S3F/D35V	9,000 ± 39	66.6
S3F/D35I	8,300 ± 32	67.8

### MD simulation

**Figure 4:**
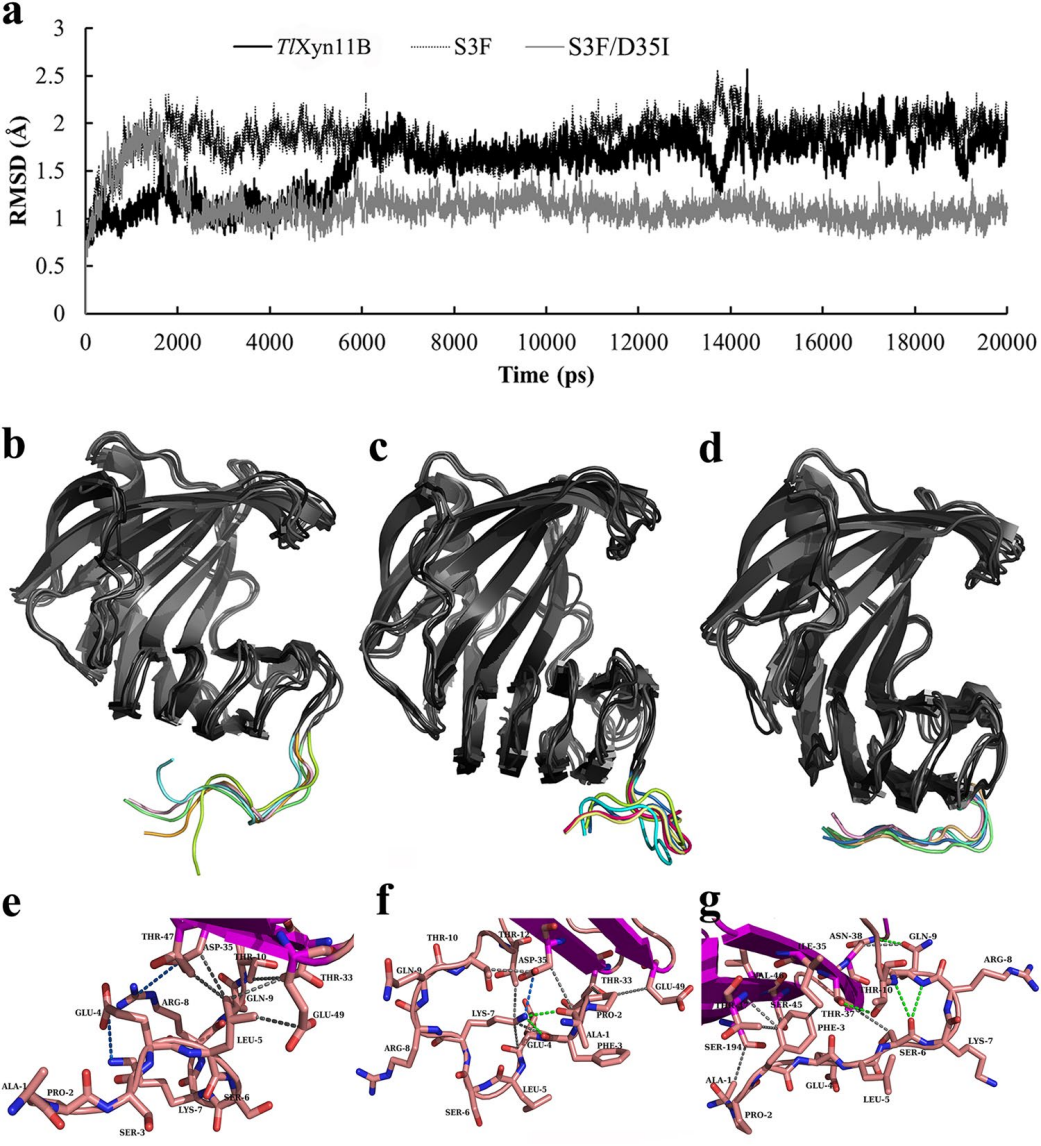
Structure analysis of *Tl*Xyn11B and its mutants S3F and S3F/D35I. (**a**) The RMSD values determined by the MD simulation. (**b**,**c**,**d**) Structure alignment of the five frames (one frame per ns in the last 5 ns) of *Tl*Xyn11B, S3F, and S3F/D35I, respectively. (**e**,**f**,**g**) Local interactions of *Tl*Xyn11B, S3F, and S3F/D35I predicted by the Ring web server. The salt bridges, hydrogen bonds and Van der Waals’s interactions are indicated by blue, green and gray lines, respectively.

MD simulation was carried out to explore the mechanism of improved thermostability of S3F and S3F/D35I. Root mean square deviation (RMSD) values of *Tl*Xyn11B, S3F and S3F/D35I were calculated with the first conformations as references, and mutant S3F/D35I showed a rather stable profile with lowered RMSD values (Fig. 4a). Structure alignment of the five frames sampled from the last 5 ns trajectory (one frame per nanosecond) indicated that the N-terminus of *Tl*Xyn11B was more flexible than those of S3F and S3F/D35I (Fig. 4b–d). Namely, the N-termini of S3F and S3F/D35I were more steadily immobilized at the bottom of the palm regions through protein inner interaction. The last frames of the trajectories of *Tl*Xyn11B and its two mutants were submitted to RING web server for amino acid interaction analysis. As shown in Fig. 4e–g, single mutant S3F demonstrated more amino acid interactions than the wild-type, while double mutant S3F/D35I formed a sandwich conformation (Ser45-Phe3-Thr47/Ile35) to fix the N-ternimus to the palm region.

## Discussion

In this study, a highly active xylanase, *Tl*Xyn11B, was identified in *T. leycettanus* JCM12802. It was acidophilic and thermophilic, showing maximal activity at pH 3.5 and 65 °C and great stability from pH 1.0 to 10.5. *Tl*Xyn11B had similar pH and temperature optima to the GH11 xylanases from *Talaromyces versatilis* ^20^ and *Penicillium occitanis* ^21^, but retained more activity at pH 2.0 and lost activity at pH 6.0 and above. These acidic xylanases are entirely different from neutral and alkaline xylanases of GH11 (optimally active at pH 6.0–8.0) in functional pH^22–25^. Although the temperature optimum (65 °C) is similar to many xylanase counterparts, its thermostability is much worse than the thermostable *Th*xyn11A^24^ and Xyn11A^26^. Moreover, the pH stability range of *Tl*Xyn11B is much broader than those of XYNF11a^23^, XynA^27^ and XynC83^28^. *Tl*Xyn11B had a specific activity of 8,400 U/mg, which is much higher than most GH11 xylanases^11,18,28,29^. In addition, its hydrolysis products were mainly xylooligosaccharides, thus *Tl*Xyn11B has potential to generate prebiotic xylooligosaccharides for feed and food purposes^30,31^.

The N-termini of GH11 xylanases play vital roles in thermostability^14,16,32^. For example, by introducing a disulfide bond, the *T*_m_ value of TLX was elevated from 66 °C to 74 °C^15^, and the XYNII from *Trichoderma reesei* had increased thermostability by 11 °C^33^. It means that the covalent interaction between the N-terminal sequence and the palm region is significant in enhancing thermostability. Therefore, a rational design of the N-terminal sequence was carried out to engineer *Tl*Xyn11B in the present study. After two rounds of site-saturated mutagenesis, double mutation S3F/D35I contributed to the enhanced ∆*T*_m_ value of 7.8 °C and improved the thermostability of *Tl*Xyn11B at 50–70 °C. The *T*_m_ value of the surface charge engineered XynA^17^ was improved from 330.4 K to 332.4 K, which was significantly lower than the improvement of *Tl*Xyn11B (333.0 to 340.8 K); on the other hand, the *Tl*Xyn11B activity (8,300 U/mg) was much higher than the mutated XynA activity (47 U/mg). Another intriguing finding is that the improved thermostability has no impairment on the enzyme activity (Table 2).

Analysis of the amino acid interactions in *Tl*Xyn11B, S3F and S3F/D35I (the latest frames of simulation trajectories) using the RING web server provides some important information (Fig. 4e). Glu4-Lys7 and Arg8-Asp35 are able to form salt bridges, while Leu5 and Thr10 tend to form Van der Waals interactions with neighboring residues like Thr33, Asp35, Thr47, and Glu49. In the mutant S3F, more interactions are detected (Fig. 4f), including hydrogen bonds formed by Ala1, Pro2, Phe3, Glu4, Lys7 and Thr10 and Van der Waals interactions or salt bridges formed by Lys7, Thr12, Asp35, Thr33, and Glu49. Although mutant S3F/D35I lost a salt bridge formed by Arg8 and Asp35 (Fig. 4g), extensive interactions are also present, i.e. van der waals interactions formed by Ala1, Phe3, Ser6, Gln9 and Thr10 and hydrogen bonds formed by Gln9, Thr10, Ile35, Thr37, Asn38, Ser45, Val46, Thr47 and Ser197. Moreover, Ser6 may form main chain-main chain hydrogen bonds with Gln9 or Thr10, Thr10 and Gln9 may form hydrogen bonds with the side chains of Thr37 and Asn38, and Thr37 and Asn38 may form Van der Waals interactions with Ser6 and Gln9. In addition, Phe3 and Ile35 may construct a sandwich structure with Ser45 and Thr47 (Ser45-Phe3-Thr47/Ile35), which might make the local conformation more rigid. These interactions altogether seem to fix the N-terminus stably. Interestingly, the wild-type and mutants S3F and S3F/D35I seemed to employ different mechanisms to confine the N-termini, which may involve different interactions of different amino acid residues.

Statistical analysis by Hotspot Wizard 2.0 indicated that the residue at position 35 is more likely to be Thr, Ser or Asn with amino frequencies of 40.5%, 33.5%, and 10.5%, respectively. Ile and Met are absent at this site, while Gln (3%) and Val (0.5%) are seldom. Similar situation occurs to Ser3, which only accounts for 5% frequency (higher than the 1% of Phe). It suggests that majority of GH11 xylanases are probably thermolabile since those thermo-related amino acids only exist in minor groups. The introduction of amino acids with low frequency (i.e. S3F and D35I) may introduce more noncovalent interactions (such as Ser45-Phe3-Thr47/Ile35) to fix the N-terminus at the protein bottom and make the whole protein structure more rigid. Therefore, this computational rational design approach is suitable for engineering local conformation with sequence diversity, such as the N-terminus and loops, in which the mutated residues fit well with adjacent amino acids.

These introduced noncovalent interactions may cause similar effects to the disulfide bond introduced in TLX^15^. As both Phe3 and Ile35 exist in GH11 xylanases at an extremely low frequency, Ser45-Phe3-Thr47/Ile35 may be a new conformation in the N-terminal stabilization. Structure search using the Dali server indicated that there was no homolog structure of the N-terminus of mutant S3F/D35I in the PDB database.

In conclusion, a highly active GH11 xylanase with broad pH stability range was identified, produced and characterized. Protein engineering of two N-terminal residues by saturated mutation was successful to produce four double mutants with improved thermostability and no activity loss. This study not only provides an excellent xylanase candidate for xyloologosaccharide production, but also verifies the efficiency of rational design approach in engineering the N-terminus for thermostability improvement.

## Materials and Methods

### Strains, media, vectors and chemicals

*T. leycettanus* JCM12802 was grown in potato dextrose Broth (PDB). *Escherichia coli* Trans1-T1 was used for gene cloning. Vector pPIC9 (Invitrogen, Carlsbad, CA) was used for gene expression by being transformed into *Pichia pastoris* GS115. Beechwood xylan was purchased from Sigma-Aldrich (St. Louis, MO). The DNA purification kit, LA Taq DNA polymerase and restriction endonucleases were purchased from TaKaRa (Otsu, Japan). The total RNA isolation system kit and T4 DNA ligase were purchased from Promega (Madison, WI). All chemicals were of analytical grade and commercially available.

### Cloning of the cDNA genes

The full-length GH11 xylanase gene, *Tlxyn11B*, was identified in the genome of *T. leycettanus* JCM12802 (the whole genome sequencing in progress). After three days’ growth in induced medium, the total RNA was extracted from the mycelia and purified using the Promega SV Total RNA Isolation System according to the manufacturer’s instructions. The total RNA was used for cDNAs synthesis *in vitro* using the ReverTra Ace-a-™ kit (TOYOBO, Osaka, Japan). The cDNAs was used as a template for PCR amplification with specific primers (Supplementary Table 2). The PCR product of the appropriate size was digested with *Eco*RI and *Not*I and ligated into the *Eco*RI-*Not*I-digested pPIC9 vector for expression.

### Sequence and structure analysis

BLASTx program (http://www.ncbi.nlm.nih.gov/BLAST/) was used to identify the cDNA sequence of *Tl*Xyn11B. Clustal Omega (http://www.ebi.ac.uk/Tools/msa/clustalo/) was used to align the nucleotide and amino acid sequences, and the results were demonstrated by ESPript 3.0 (http://espript.ibcp.fr/ESPript/cgi-bin/ESPript.cgi). Proparam tool (http://web.expasy.org/protparam/) was used to predict the molecular mass and theoretical isoelectric point (*p*I). SignalP4.0 server (http://www.cbs.dtu.dk/services/SignalP/) was used to predict the signal peptide. Modeller 9.13 was used to build the homology model of *Tl*Xyn11B with Chimera 1.10 (http://www.cgl.ucsf.edu/chimera/) to process energy minimization. NetNGlyc 1.0 server (http://www.cbs.dtu.dk/services/NetNGlyc/) was applied to predict the potential *N*-glycosylation site. Pymol 0.99rc was employed to represent the homology model and aligned structures. The catalytic amino acid residues, amino acids frequency and high β-factor sites were predicted by Hotspot Wizard^34^ (http://loschmidt.chemi.muni.cz/hotspotwizard/). Amino acid interactions were predicted by Residue Interaction Network Generator (RING) web server^35^ (http://protein.bio.unipd.it/ring). Dali server (http://ekhidna2.biocenter.helsinki.fi/dali/) was used to search similar structures in the PDB database.

### Heterologous expression and purification *Tl*Xyn11B

The recombinant plasmid pPIC9-*Tlxyn11B* was linearized using *Bgl*II and then transformed into *P. pastoris* GS115 competent cells by electroporation. The positive transformants were screened via enzymatic activity measurement in shake tubes, and the transformant with the highest xylanase activity was cultivated for fermentation in a 1-L Erlenmayer flask as described by Luo *et al*.^36^.

Cells were removed by centrifugation, and the culture supernatants were subject to concentration through a Viva flow 200 ultrafiltration membrane (cutoff 10 kDa; Vivascience, Hannova, Germany). The crude enzyme was dialyzed in 20 mM citric acid-Na_2_HPO_4_ (buffer A, pH 7.2) at 4 °C overnight and loaded onto a HiTrap Q Sepharose XL 5 mL FPLC column (GE Healthcare, Uppsala, Sweden) equilibrated with buffer A. A linear gradient of NaCl (0–1.0 M) was used to elute the proteins. Fractions bearing xylanolytic activity were pooled, dialyzed in buffer A, and concentrated by ultrafiltration at 4,000 × *g* for 40 min at 4 °C using an Amicon Ultra Centrifugal Filter Device PL-10 (Millipore, Billerica, MA). The fractions exhibiting enzyme activities were pooled and assayed by sodium dodecyl sulfate-polyacrylamide gel electrophoresis (SDS-PAGE). The purified recombinant *Tl*Xyn11B was deglycosylated by endo-β-N-acetylglucosaminidase H (Endo H) (New England Biolabs, Hitchin, UK) at 37 °C overnight. The protein concentration was determined using the NanoVue Plus device (GE Healthcare, Uppsala, Sweden) and Lowry protein assay^37^, with bovine serine albumin as the standard.

### MALDI-TOF/TOF MS analysis

MS and MS/MS data for protein identification were obtained by using a MALDI-TOF-TOF instrument (4700 proteomics analyzer; Applied Biosystems, Foster City, CA). The MS spectra were recorded in reflector mode in a mass range from 800 to 4,000 with a focus mass of 2,000. The TOF/TOF calibration mixtures (AB SCIEX) were used to calibrate the spectrum to a mass tolerance within 10 ppm. For MS calibration, autolysis peaks of trypsin ([M + H] + 842.5100 and 2,211.1046) were used as internal calibrates, and the most intense ion signals (up to 10) were selected as precursors for MS/MS acquisition, excluding the trypsin autolysis peaks and the matrix ion signals.

Peptide mass finger printing PMF and MS/MS queries were performed by using the MASCOT search engine 2.2 (Matrix Science, London, UK) embedded into GPS-Explorer Software 3.6 (Applied Biosystems). A GPS Explorer protein confidence index ≥95% were used to align with the deduced protein sequence of *Tl*Xyn11B.

### Biochemical characterization of purified recombinant *Tl*Xyn11B

The standard assay for xylanase activity was performed at 65 °C for 10 min in citric acid-Na_2_HPO_4_ (pH 3.5; 200 mM) containing 1.0% (w/v) beechwood xylan. The amount of reducing sugar released was determined using the 3,5-dinitrosalicylic acid (DNS) method^38^. For assays of pH adaptability and stability, glycine-HCl (pH 1.0–2.5; 100 mM), citric acid-Na_2_HPO_4_ (pH 2.5–8.0; 200 mM) and glycine-NaOH (pH 8.0–11.0; 100 mM) were used. The optimal pH for *Tl*Xyn11B activity was determined at 65 °C and pH 1.5 to 6.0 for 10 min. The pH stability of *Tl*Xyn11B was determined by pre-incubating the enzyme (diluted 20 times with buffers of pH 1.0 to 11.0, total volume of 1 mL) at 37 °C for 1 h without substrate and measuring the residual activities under standard conditions (pH 3.5 and 65 °C for 10 min). The optimal temperature for *Tl*Xyn11B activity was determined by performing the activity assay at 30 to 80 °C and at pH 3.5 for 10 min. Thermal stability was tested by pre-incubating the enzyme (approximately 50 μg/mL in 200 mM citric acid-Na_2_HPO_4_, pH 5.5, total volume of 1 mL) at 50–70 °C for 30 min without substrate and monitoring the residual xylanase activities under standard conditions.

The effect of different metal ions and chemical reagents on the *Tl*Xyn11B activity was determined in the presence of 1, 5 and 10 mM of NaCl, KCl, LiCl, MgSO_4_, CaCl_2_, HgSO_4_, AgNO_3_, ZnSO_4_, FeCl_3_, NiSO_4_, CuSO_4_, SDS and EDTA. The reaction systems without any chemical addition were treated as the control.

The *K*_*m*_ and *V*_*max*_ values of *Tl*Xyn11B were determined under standard conditions for 5 min with 1−10 mg/mL beechwood xylan as the substrate. The GraphPad Prism version 5.01 (La Jolla, CA) was used for data analysis with the Michaelis-Menten model. Each experiment was repeated three times.

### Analysis of hydrolysis products

One milliliter of citric-Na_2_HPO_4_ buffered beechwood xylan (pH 3.5) was treated by 2 U of *Tl*Xyn11B at 50 °C overnight. The enzyme was then removed from the reaction system through the Nanosep centrifugal 3 K device (Pall, New York, NJ). Aliquots (100 μL) of 100-fold diluted hydrolysate samples were analyzed by high-performance anion-exchange chromatography (HPAEC-PAD) (Dionex, Sunnyvale, CA) equipped with a pulsed amperometric detector ICS-5000, a CarboPac PA100 guard column (4 × 50 mm) and an analytical column (4 × 250 mm). The xylo-oligosaccharides were eluted by 100 mM NaOH at the rate of 1 mL/min and at the temperature of 22 °C. Xylose, xylobiose, xylotriose, xylotetrose, xylopentose and xylohexose were used as the standards. Each experiment was repeated three times.

### Site-directed mutagenesis and mutant characterization

S3 was subject to saturated mutation. After thermostability screening in 96-well microplates, the mutant with greatest thermostability was selected for the second saturation mutation at D35. Mutants harboring different mutation sites were generated using overlap PCR with KOD neo Plus polymerase (TOYOBO, Osaka, Japan) and specific primers (Supplementary Table 2). The PCR products were sequenced for verification. Heterologous expression and characterization of mutant enzymes were conducted as described for the wild-type. And the correct mutants were further confirmed by PCR amplification with the AOX primers.

### Differential scanning calorimetry (DSC) analysis

The melting temperatures (*T*_m_) of *Tl*Xyn11B and its mutants were analyzed by using the DSC. The proteins were diluted in 10 mM citric acid-Na_2_HPO_4_ (pH 6.0) to approximately 0.25 mg/mL, and heated over the temperature range of 30 to 100 °C at a rate of 1 °C/min in Nano-DSC (TA Instruments, New Castle, DE). The experiment was repeated twice.

### Molecular dynamics (MD) simulation

Amber 14 package was used to carry out the MD simulation analysis at a temperature of 310 K for a 20 ns process with force field ff99SB. The protein was placed in a truncated octahedron with solvate model TIP3P. The closest distance between protein atom and the periodic box was set to 12 angstroms, while the time step was set to 2 fs. Before the simulation, the system was firstly energy-minimized by steepest descent methods (maximum steps of 40,000) and conjugate gradient method (maximum steps of 1,000) with C_α_ restrained; then the energy minimization was repeated without atom restraint; and finally this system was heated from 0 K to 310 K within 35 ps before the MD simulation. Trajectory data were then analyzed using the CPPTRAJ software^39^.

### Data availability statement

The datasets generated and analyzed during the current study are available from the corresponding author on reasonable request.

## Electronic supplementary material

Supplementary material

## References

[1] Berrin J-G, Juge N (2008). Factors affecting xylanase functionality in the degradation of arabinoxylans. Biotechnol. Lett.

[2] Juturu V, Wu JC (2012). Microbial xylanases: Engineering, production and industrial applications. Biotechnol. Adv..

[3] Collins T, Gerday C, Feller G (2005). Xylanases, xylanase families and extremophilic xylanases. FEMS Microbiol. Rev..

[4] Henrissat B, Bairoch A (1996). Updating the sequences-based classification of glycosyl hydrolases. Biochem. J..

[5] Bottcher D, Bornscheuer UT (2010). Protein engineering of microbial enzymes. Curr. Opin. Microbiol..

[6] Otten LG, Hollmann F, Arends IWCE (2010). Enzyme engineering for enantioselectivity: from trial-and-error to rational design?. Trends Biotechnol..

[7] Lehmann M, Wyss M (2001). Engineering proteins for thermostability: the use of sequence alignments versus rational design and directed evolution. Curr. Opin. Biotechnol..

[8] Petrik ID, Liu J, Lu Y (2014). Metalloenzyme design and engineering through strategic modifications of native protein scaffolds. Curr. Opin. Chem. Biol..

[9] Damborsky J, Brezovsky J (2014). Computational tools for designing and engineering enzymes. Curr. Opin. Chem. Biol..

[10] Damásio ARDL (2011). Heterologous expression of an *Aspergillus niveus* xylanase GH11 in *Aspergillus nidulans* and its characterization and application. Process Biochem..

[11] Singh RK (2013). Molecular cloning and characterization of a GH11 endoxylanase from *Chaetomium globosum*, and its use in enzymatic pretreatment of biomass. Appl. Microbiol. Biotechnol..

[12] Xiao Z, Grosse S, Bergeron H, Lau PCK (2014). Cloning and characterization of the first GH10 and GH11 xylanases from *Rhizopus oryzae*. Appl. Microbiol. Biotechnol..

[13] Gao SJ (2013). Engineering hyperthermostability into a mesophilic family 11 xylanase from *Aspergillus oryzae* by in silico design of N-terminus substitution. Biotechnol. Bioeng..

[14] Zhang H, Li J, Wang J, Yang Y, Wu M (2014). Determinants for the improved thermostability of a mesophilic family 11 xylanase predicted by computational methods. Biotechnol. Biofuels.

[15] Wang Y (2012). Improved thermal performance of *Thermomyces lanuginosus* GH11 xylanase by engineering of an N-terminal disulfide bridge. Bioresour. Technol.

[16] Zhang S (2010). Five mutations in N-terminus confer thermostability on mesophilic xylanase. Biochem. Biophys. Res. Co.

[17] Alponti JS, Maldonado RF, Ward RJ (2016). Thermostabilization of *Bacillus subtilis* GH11 xylanase by surface charge engineering. Int. J. Biol. Macromol..

[18] Singh RK (2012). Molecular cloning and characterization of a GH11 endoxylanase from *Chaetomium globosum*, and its use in enzymatic pretreatment of biomass. Appl. Microbiol. Biotechnol..

[19] Mahanta P, Kumar K, Reddy VS, Ramakumar S (2015). Structural insights into N-terminal to C-terminal interactions and implications for thermostability of a (β/α)_8_-triosephosphate isomerase barrel enzyme. FEBS J..

[20] Lafond M, Guais O, Maestracci M, Bonnin E, Giardina T (2014). Four GH11 xylanases from the xylanolytic fungus *Talaromyces versatilis* act differently on (arabino)xylans. Appl. Microbiol. Biotechnol..

[21] Driss D, Bhiri F, Siela M, Ghorbel R, Chaabouni SE (2012). Purification and properties of a thermostable xylanase GH 11 from *Penicillium occitanis* Pol6. Appl. Biochem. Biotechnol..

[22] Amaya-Delgado L (2010). Cloning and expression of a novel, moderately thermostable xylanase-encoding gene (*Cf*xyn11A) from *Cellulomonas flavigena*. Bioresour. Technol.

[23] Jeya M, Thiagarajan S, Lee JK, Gunasekaran P (2009). Cloning and expression of GH11 xylanase gene from *Aspergillus fumigatus* MKU1 in *Pichia pastoris*. Biosci. Bioeng.

[24] Jin C (2011). Cloning, expression, and characterization of an alkaline thermostable GH11 xylanase from. Thermobifida halotolerans YIM 90462 T. Bioresour. Technol.

[25] Zhou J (2011). Symbiotic *Streptomyces* sp. TN119 GH 11 xylanase: a new pH-stable, protease- and SDS-resistant xylanase. J. Ind. Microbiol..

[26] Qiao W (2014). Biochemical characterization of a novel thermostable GH11 xylanase with CBM6 domain from *Caldicellulosiruptor kronotskyensis*. J. Mol. Catal. B Enzym.

[27] Wei Q, Shao W (2011). Cloning, expression and characterization of glycoside hydrolase family 11 endoxylanase from *Bacillus pumilus* ARA. Biotechnol. Lett..

[28] Zhao L (2013). Two family 11 xylanases from *Achaetomium* sp. Xz-8 with high catalytic efficiency and application potentials in the brewing industry. J. Agric. Food Chem..

[29] Wei F, Gao H, Cao Y, Shan A (2014). Cloning and expression of a xylanase xyn B from *Aspergillus niger* IA-001 in *Pichia pastoris*. J. Basic Microbiol..

[30] Maesschalck CD (2015). 2015. The effects of xylo-oligosaccharides on performance and microbiota in broiler chickens. Appl. Environ. Microbiol.

[31] Ma R (2017). Utility of thermostable xylanases of Mycothermus thermophilus in generating prebiotic xylooligosaccharides. J. Agric. Food Chem..

[32] Zhang S, He Y, Yu H, Dong Z (2013). Seven N-terminal residues of a thermophilic xylanase are sufficient to confer hyperthermostability on its mesophilic counterpart. PLoS ONE.

[33] Fenel F, Leisola M, Janis J, Turunen O (2004). A de novo designed N-terminal disulphide bridge stabilizes the *Trichoderma reesei* endo-1,4-β-xylanase II. J. Biotechnol..

[34] Bendl J (2016). HotSpot Wizard 2.0: automated design of site-specific mutations and smart libraries in protein engineering. Nucleic Acids Res.

[35] Damiano P, Giovanni M, Tosatto SCE (2016). The RING 2.0 web server for high quality residue interaction networks. Nucleic Acids Res.

[36] Luo H (2009). A thermophilic and acid stable family-10 xylanase from the acidophilic fungus *Bispora* sp. MEY-1. Extremophiles.

[37] Lowry OH, Rosebrough NJ, Farr AL, Randall RJ (1951). Protein measurement with the Folin phenol reagent. J. Biol. Chem..

[38] Miller GL (1959). Use of dinitrosalicylic acid reagent for determination of reducing sugar. Anal. Chem..

[39] Roe DR, Rd CT (2013). PTRAJ and CPPTRAJ: Software for processing and analysis of molecular dynamics trajectory data. J. Chem. Theory Comput.

