# Supplementary material for: Thermostability improvement of a Talaromyces leycettanus xylanase by rational protein engineering
Source: Sci Rep. 2017 Nov 10;7:15287. doi: 10.1038/s41598-017-12659-y (PMC5681632; doi:10.1038/s41598-017-12659-y)
Supplement: Supplementary file 1 — Supplementary material [file 41598_2017_12659_MOESM1_ESM.pdf]

Supplementary materials to

Thermostability improvement of a *Talaromyces leycettanus* xylanase by semi-rational protein engineering

Xiaoyu Wang[a,b] · Rui Ma[a] · Xiangming Xie[a] · Weina Liu[a] · Tao Tu[a] · Fei Zheng[a,b] · Shuai You[a] · Jianzhong Ge[a] · Huifang Xie[a] · Bin Yao[a,*] · Huiying Luo[a,*]

Key Laboratory for Feed Biotechnology of the Ministry of Agriculture, Feed Research Institute, Chinese Academy of Agricultural Sciences, Beijing 100081, People's Republic of China [a]; College of Biological Sciences and Biotechnology, Beijing Forestry University, Beijing 100083, People's Republic of China [b]

Corresponding authors. E-mail: binyao@caas.cn; luohuiying@caas.cn.

Supplementary Table 1 Summary of the recombinant *Tl*Xyn11B purification

Purification step	Protein concentration (μg/mL)	Total activity (U)	Specific activity (U/mg)	Recovery rate (%)
Crude enzyme	86	340,600	6,600	-
Vivaflow	532	180,900	6,800	53
Anion exchange	384	164,300	8,600	48

Supplementary Table 2 Primers used in this study

Primers	Sequences (5′→3′)[a]
*Tl*Xyn11BF	ATGGTGTCCTTCTCCTCCCTCGC
*Tl*Xyn11BR	CTAGGAAACAGTAATGGAAGAGGACCCG
*Tl*Xyn11B_EcoF	ACGAATTCGCTCCTAGCGAGCTCTCCAAACGC
*Tl*Xyn11B_NotR	ATTTGCGGCCGCCTAGGAAACAGTAATGGAAGAGGACCCG
*Tl*Xyn11B_T1-F	ACGAATTCCGCCAAACACTAACCTCAAGCTCC
*Tl*Xyn11B_T2-F	ACGAATTCACACTAACCTCAAGCTCCACTGGC
*Tl*Xyn11B_S3-F	ACGAATTCGCTCCTNNKGAGCTCTCCAAACGC
*Tl*Xyn11B_D35-F	GGAACGGTCNNKTACACTAATGGCAA
*Tl*Xyn11B_D35-R	ATTGCCATTAGTGTAMNNGACCGTTCC
5sAOX	TTGCGACTGGTTCCAATTGACAAGCTTTTG
3sAOX	TCCTCTTGATTAGAATCTAGCAAGACCGGTCTTC

[a] The restriction sites are underlined

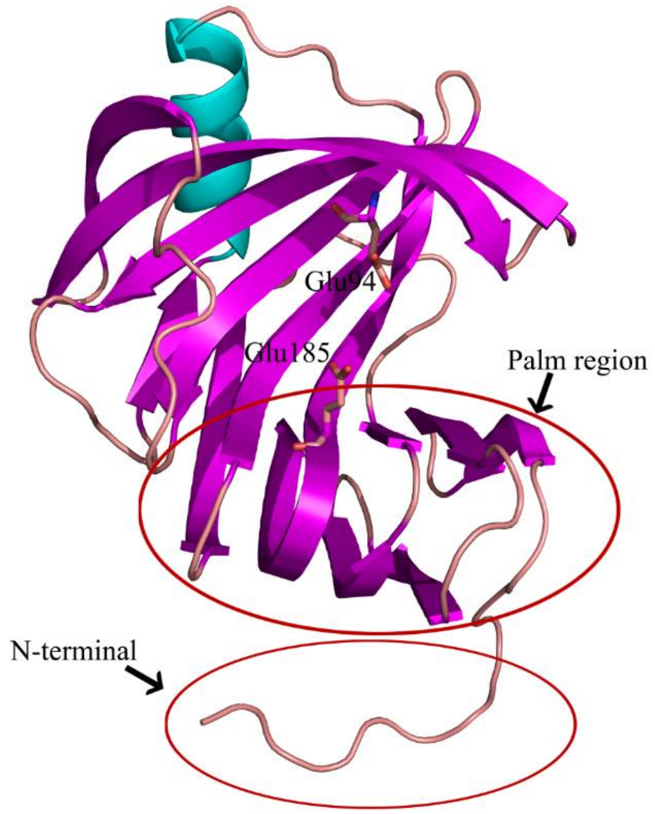

Supplementary Fig. 1 Homology modeling of *Tl*Xyn11B with the GH11 endo-1,4-β-xylanase from *T. cellulolyticus* (PDB: 3WP3) as template. The catalytic residues, palm region and N-terminus are indicated.

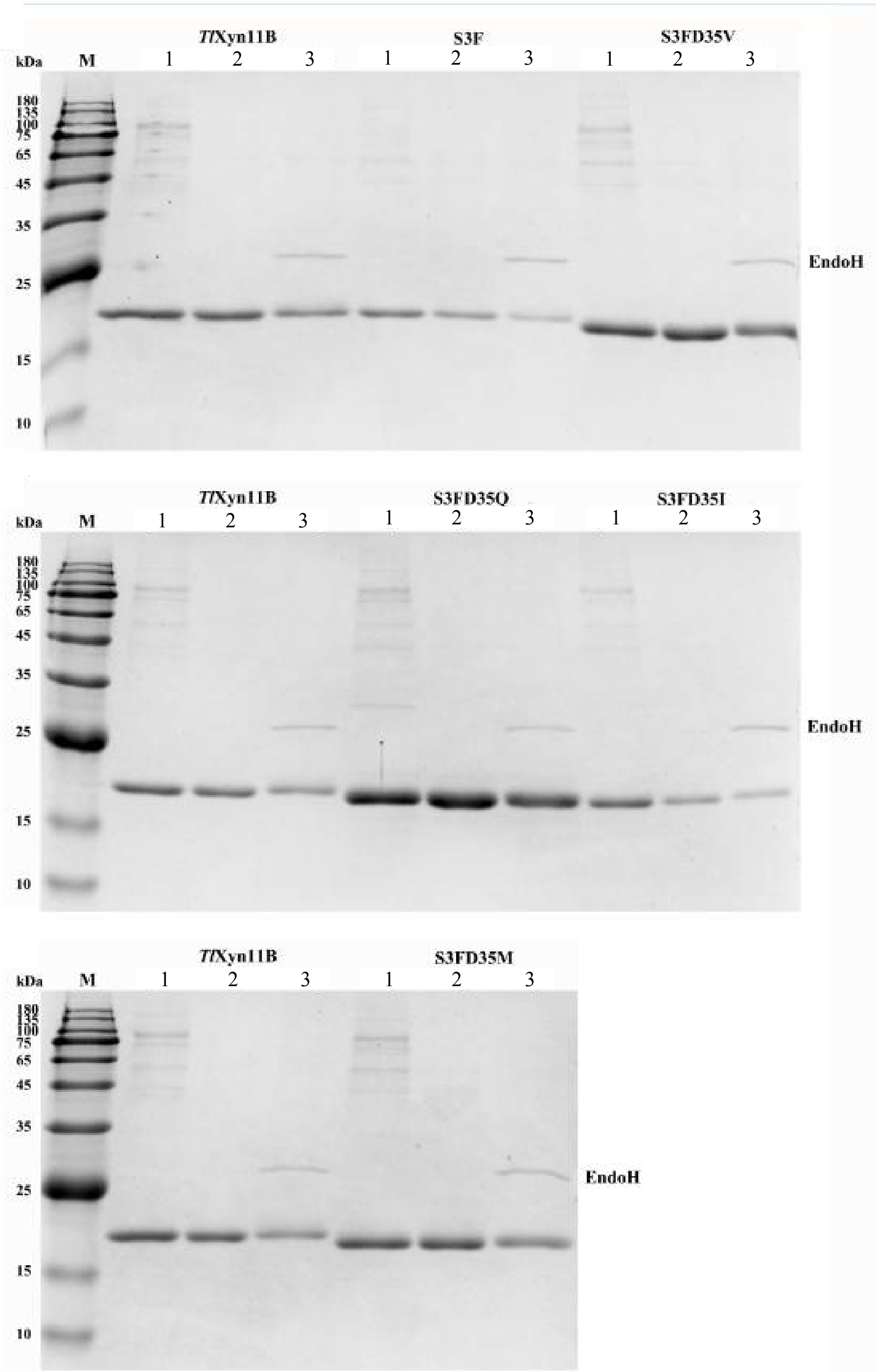

Supplementary Fig. 2 SDS-PAGE analysis of *Tl*Xyn11B and its mutants. Lanes: M, the molecular mass standards; 1, the crude enzymes; 2, the purified recombinant enzymes; 3, the deglycosylated enzymes with Endo H treatment.

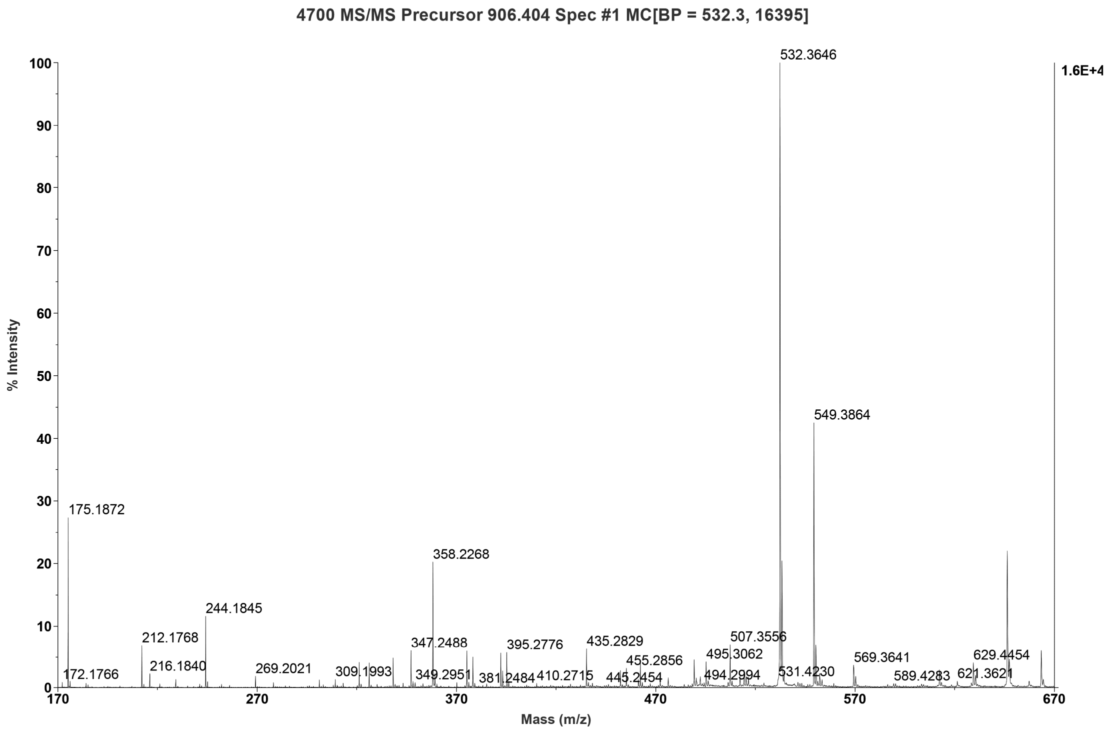

Supplementary Fig. 3 MS identification of the purified recombinant *Tl*Xyn11B.
